# Plastic Filling

**Published:** 1859-08

**Authors:** 


(From the Dental News Letter.)

### PLASTIC FILLING.

MESSRS. EDITORS:—You are aware that some three years since, I was engaged in a series of experiments with oxy-chloride of zinc. I think I promised you, under certain contingencies, I would furnish for the *News Letter* a history of my experiments. I now proceed to redeem my promise.

I was induced to experiment with zinc and its compounds by meeting with the following extract from "*The Chemist.*"

"This cement is a basic oxy-chloride of zinc; it is formed by moistening oxide of zinc with the liquid chloride of zinc, iron, manganese, nickel or cobalt. These chlorides may be replaced by chlorodic acid. The hardness of the cement is proportinate to the concentration of the chloride and the density of the oxide. Washed residues arising from the manufacture of white zinc are employed, or ordinary white zinc may be calcined to redness. Chloride of zinc, marking from 50 to 60 degrees of Baume's areometer, is used, and in order that the cement may set less quickly, about three per cent. of borax or sal ammoniac is dissolved in the oxide, or else the oxide is calcined after being moistened with water containing a little borax. The mastic or cement obtained by the combination of the above substances may be run into moulds like plaster; it is as hard as marble; cold moisture, and even boiling water, are without action upon it. It resists 576 degrees Fahrenheit, and even the most powerful acids attack it but very slowly. The new plastic matter is not expensive, and its cost may be still considerably diminished by mixing with the oxide of zinc, metallic, silicious, or calcareous matters, such as iron filings or borings, iron pyrites, blonde, emery, granite, marble, or any hard calcareous matters.—Soft matters, as chalk, etc., will not do. The highest and most varied colors may be given to the cement, which makes it suitable for tables and mosaic pavement of great hardness and beauty. It may also be used for making moulded objects of art, such as statutes, medallions, bas-reliefs, etc. It is also well suited for ceilings, and several good dentists of Paris have employed it successfully for several years for filling decayed teeth, and even for cementing the parts of a set of teeth."

I commenced operations with the common commercial oxide of zinc, and such liquid chloride of zinc as I could obtain at the druggists, but soon found them utterly worthless. I then tried calcining the oxide and after a great number of efforts, I succeeded in obtaining an article which, when mixed with a highly concentrated chloride of zinc, (prepared by myself,) would harden, without shrinking, into a very solid mass. By varying the process of calcination, I could at pleasure, produce an oxide that would harden in from half a minute to an hour or more. While plastic, it could with great ease, be introduced into the cavity of a tooth, and if it had been properly manipulated by the time the plug was smoothed and trimmed, it would be hard enough to be entirely uninjured by the fluids of the mouth. I pluged several teeth with it, and had one of my own filled with it. I could, with different metallic pigments, vary the color till it could not be distinguished from the substance of the tooth filled. After observing the appearance of the plug from day to day, and finding no appreciable change, I was delighted with the result of my experiments and was almost ready to issue circulars to the profession, announcing the discovery of a plastic material perfectly suited to the wants of dentists, for filling teeth. I also used it for making continuous gums on gold and silver plates, for which it seemed admirably adapted.

I think it is Horace who recommends that young authors should not publish their works until they shall have been written nine years. I remembered that; and, although it was very trying to me to defer reaping my harvest glory until time should prove the worth or worthlessness of my discovery, I nevertheless did manfully combat my thirst for fame, and waited till I found that my material did not make an enduring highway for that old traveler Time. I found it gradually disappearing under his heel; the durability of it varying very much in different mouths. In some cases, large plugs of it would disappear in six months or less. In my own mouth, although the plug is not large and has been in over three years, yet it is not half dissolved; but mine, I believe, is the most favorable case in all my experiments.

Although I regard my efforts to find a plastic, suitable for plugging teeth, as next thing to a total failure, (for its legitimate use in dentistry is reduced to an exceedingly narrow field,) yet I regard the oxy-chloride of zinc as peculiarly adapted to a great variety of useful purposes in the arts; and as a knowledge of it is extended, it will be more and more highly appreciated. It is to be hoped that the value of a properly prepared article in the art, may soon be more generally known. A Friend to Progress.

